# The Effect of Pharyngeal Packing during Nasal Surgery on the Incidence of Post Operative Nausea, Vomiting, and Sore Throat

**Published:** 2014-10

**Authors:** Ali Karbasforushan, Behzad Hemmatpoor, Behnam Reza Makhsosi, Tayebeh Mahvar, Parisa Golfam, Behrooz Khiabani

**Affiliations:** 1 *Department of Anesthesiology, Emam Reza Hospital, Kermanshah University of Medical Sciences, Kermanshah, Iran.*; 2 *Departments ofSurgery, Emam Reza Hospital, Kermanshah University of Medical Sciences, Kermanshah, Iran.*; 3 *M.Sc Degree in nursing (Medical-Surgical), Taleghani HospitalKermanshah University of Medical Sciences, Kermanshah, Iran.*; 4 *Member of Iranian Epidemiologist Association.*

**Keywords:** Nasal surgery, Nausea and vomiting, Throat pain

## Abstract

**Introduction:**

Nausea and vomiting after ear, nose and throat (ENT) surgery is one of the most common and notable problems facing anesthesiologists in this area. This study was conducted to determine the effect of a pharyngeal pack on the severity of nausea, vomiting, and sore throat among patients after ear, pharynx, and throat surgeries.

**Materials and Methods:**

This randomized clinical study was performed in 140 patients (61 men and 79 women; age range, 20–40 years) who had undergone nasal surgery in 2010. Patients were divided into two groups: the first group were treated using a pharyngeal pack (case group) and the second group were managed without a pharyngeal pack (control group). Statistical analysis was performed using the Chi-square test and the Mann-Whitney U test. SPSS software was used for data analysis.

**Results:**

The mean severity of nausea and vomiting in the two groups was 2.057, 1.371 and 1.100, respectively, with no significant differences between groups. However, the mean severity of sore throat was 1.714 in the group with the pharyngeal pack and 1.385 in the group without pharyngeal pack (P=0.010).

**Conclusion:**

Not only does a pharyngeal pack in ENT surgery not reduce the extent and severity of nausea and vomiting, but it also increases the severity of sore throat in patients when leaving the recovery room and discharging hospital.

## Introduction

Nausea is a subjective and non- visible phenomenon resulting from an unpleasant feeling in the back of the pharynx and epigastria which evokes the urgent need for vomiting in the patient and might lead to pressure-associated withdrawal of stomach contents out of the mouth. Postoperative nausea and vomiting (PONV) is a significant problem and is the most common complication facing anesthesiologists. This complication results in a delay in the release of approximately 30% of patients from hospital (1). Despite the large sums of money that patients devote to buying anti-vomiting drugs, 25% of patients experience nausea and vomiting for up to 24 hours after surgery (2). The annual cost of treating the PONV is nearly a $100 million.  Improving understanding of PONV as well as managing the condition and preventing serious side effects have been the subject of many studies (3). Consequences of nausea and vomiting after surgery, such as pulmonary aspiration, dehydration, electronic disturbances and injury in the place of surgery, as well as the impact on the cost of treatment, can increase anxiety and dissatisfaction with the surgery performed (4).

Since the return of blood and gastric secretions is one of the factors that causes nausea and vomiting after bleeding from surgery, including nose surgery, anesthesiologists believed that creating a dam against the entry of blood from the throat into the stomach would prevent this complication to some extent.

The result of various studies have failed to prove this, and indicate that a lower pharyngeal pack does not reduce PONV, and increases the rate of sore throat. Furthermore, the results of some studies show that the use of gas dipped in paraffin wax reduces sore throat, while another study states that a lower pharyngeal pack in patients undergoing surgery of the jaw and face has no effect on postoperative sore throat (6,10,11). The aim of this study was to compare the impact of a lower pharyngeal pack on PONV and sore throat following surgery of the nose.

## Materials and Methods

This study is the result of a research plan approved by the Medical Sciences and Health Services of Kermanshah (Contract number: 347, dated April 24, 2009). This was a clinical trial in 140 patients aged 20–40 years performed in the hospital of Imam Reza (A.S.) Kermanshah in 2010. Sampling was performed using a random method. Prior to the study, informed consent was obtained from all patients. Exclusion criteria included patients with thyroid dysfunction, hypertension, nausea and vomiting; drug users; severe hemorrhagic disease; diabetes; digestive disorders; migraines; anti-acid medication consumers; and patients with pathology of the lung and pharynx. Data were collected by a questionnaire, checklist of complications occurrence, and information collection. The scientific validity of the questionnaire was measured through the content validity; its scientific reliability was measured through concurrent observation. The scientific reliability of the form used for recording scientific information related to the severity, as well as the checklist of complication occurrence, was conducted through concurrent observation of nausea and vomiting based on a visual analog scale (VAS) by one of the researchers and one of the nursing staff employed in the ward and an anesthesia technician. Patients were divided into two groups of 70 persons; the first group (with pharyngeal pack) and the second group (non-pharyngeal pack) were in Class 1,2, according to the American Society Anesthesiology (ASA).

In both groups, after measuring and recording baseline hemodynamic parameters (blood pressure and pulse) and pulse oximetry, and after an electrocardiogram (ECG) performed using monitoring devices, patients were similarly were hydrated using Ringer 500 ml.

After injection of midazolam (2 mg/kg) and fentanyl(2 µg/kg), induction of anesthesia was performed using sodium thiopental (4 mg/kg) and atracurium (0.5 mg kg). After intubation and ensuring proper placement of an endotracheal tube and fixing it with a wet gauze soaked with normal saline in the first group, a pharyngeal pack was positioned by one of the investigators so that the corners of the gauze were outside the patient’s lips.

The pack was inserted in the first group. The ventilation continued with a positive pressure and a solid volume of 10 ml/kg and 10 breaths per minute; and anesthesia was maintained with an isoflurane gas mixture O_2_ = 50% and N_2_O = 50%.

If control of hypotension was necessary, remifentanil was used at an infusion dose of 0/1 µg, if there were more than two muscular responses during the surgery, atracurium 0.1 mg/kg was injected using the nerve stimulation muscle response. 

Following surgery, and after suctioning the mouth, the pharyngeal pack was removed gently. Based on TOF criteria, if there were three or four muscle responses, patients were injected with neostigmine 0.2 mg and atropine 0.4 mg to restore their muscle strength.

After the patient was fully awake, the tracheal tube was removed and the patient was transferred to the recovery room. During the recovery period, the severity of nausea and vomiting and sore throat were measured by an anesthesia technician; then, after transferring to the ward, the above symptoms were measured by the nurse within the first 4 hours of arrival and at the time of discharge.

Measurement of the severity of nausea and vomiting, and sore throat was based on a VAS in which ratings are divided into four categories: zero (no symptoms), 1–3 (low symptoms), 4–7 (medium symptoms), 7 (severe symptoms). Pre-training of the patient and how to measure symptoms and signs of anesthesia technicians and nurses were provided. The required training on the expression of the above symptoms was presented to patients, while information on how to measure and record the symptoms was given to the anesthesia technician and nurse in the ward. 

Finally, all data, including pulse rate, blood pressure, nausea, vomiting and sore throat during the desired times were calculated and analyzed using means, percentages, and analytical statistics (Chi-square test and **Mann-Whitney test**) using SPSS14 software. The **significance level** of the test was P<0.05.

## Results

The mean age was 23.71±5.36 years in the pharyngeal-pack group and 24.68±5.20 years in the non- pharyngeal-pack group (Table. 1). In the pharyngeal-pack group, 32 persons (45.7%) were men and 38 persons (54.3%) were women; however in the non-pharyngeal-pack group 29 persons (41.4%) were men and 41 persons (58.6%) were women (Table. 2). Statistical analysis shows that there was a statistically significant difference between sore throat and time to enter the recovery ward in the pack group (P=0.001) (Table. 3). 

**Table 1:** Mean of age in two groups

**Group **	**N**	**Mean**	**Standard** **Deviation**
With pack	70	23.71	5.38
Without pack	70	24.68	5.20

**Table 2:** Sex of participation in two groups

**Group**	**Men**	**Women**	**Total**
With pack	32	38	70
Without pack	29	41	70

**Table 3:** Correlation between times after surgery

**Group**	**Enter to recover** **Ward(T1)**	**After 4 hour(T2)**	**Discharge(T3)**
With pack	2/057	1/371	1/10
Without pack	1/975	1/412	1/175

The mean severity of nausea and vomiting ((Enter to recover Ward (T1), After 4 hour(T2), Discharge(T3)) in the two groups was 2.057, 1.371 and 1.100, respectively; with no significant differences between groups. However, the mean severity of sore throat in the group with the pharyngeal pack was 1.714, and in the group without the pharyngeal pack was 1.385 (P=0.010). Also, the mean severity of sore throat was 1.433 in patients with the pharyngeal pack and 1.262 in the group without the pharyngeal pack (P=0.026).

## Discussion

It can be concluded that the use of a pharyngeal pack does not reduce the amount of PONV in nasal surgery, but has a significant impact on the degree of sore throat during the first 4 hours after arrival on the ward and during the discharge of the patient .control hypotension was confounder variable which can reduce bleeding; the incidence of nausea and vomiting may be due to other causes like blood entering the stomach.

In the case of throat pain, there was a direct impact of the pharyngeal pack, possibly explained by irritation on removal. Pain due to residual effects of the anesthetic drugs and the use of surgical instruments and pharyngeal pack suction are equivalent between the two treatment groups in the study. However, over time, the impact of these items is reduced and residual pain can be attributed to use of the pharyngeal pack in the case (with pack) group. The impact of a pharyngeal pack on nausea and vomiting was investigated in the following studies.

In a study by Piltcher et al.  (2007) investigating the influence of the pharyngeal pack on  nausea and vomiting and need for medications to address nausea, vomiting, and  sore throat in approximately 144 patients who underwent nasal surgery, there was no significant difference between the case and control groups in terms of the above (7).

In a study by Basha* et al.* (2007) in 100 patients undergoing nasal surgery under the influence of a pharyngeal pack, it was shown that the pharyngeal pack does not reduce the amount of nausea and vomiting, but increases sore throat (8). In a study concluded by the Fine et al. (1988) investigating the impact of a pharyngeal pack on postoperative nausea and vomiting and sore throat, it was concluded that the pharyngeal pack has the greatest influence on the occurrence of postoperative sore throat, followed by the size of endotracheal tube and its cuff volume (9). 

For further studies, it is suggested that the impact of the pharyngeal pack on other surgery with different length of operation should be examined.

## Conclusion

Not only does a pharyngeal pack in ENT surgery not reduce the extent and severity of nausea and vomiting, but it also increases the severity of sore throat in patients when leaving the recovery  room and discharging hospital.
